# Social support and financial status are associated with depression symptoms among healthcare workers one year after the onset of the COVID-19 pandemic in France

**DOI:** 10.1192/j.eurpsy.2025.1381

**Published:** 2025-08-26

**Authors:** C. Vincent, H. Scarlett, M. Melchior, C. Vuillermoz

**Affiliations:** 1 Team of social epidemiology, Institut Pierre Louis d’Epidémiologie et de Santé Publique; 2 Inserm; 3 Institut Pierre Louis d’Epidémiologie et de Santé Publique, Paris; 4 Université de Versailles, Saint-Quentin-en-Yvelines, France

## Abstract

**Introduction:**

One year after COVID-19’s emergence, studies show healthcare workers (HCWs) face significant depression risk linked to pandemic stressors. While the pandemic impacted social and economic factors, the link between depression and social support/financial hardship in HCWs remained unclear.

**Objectives:**

This study leaded in France investigates depression prevalence and its association with these factors among HCWs one year after the onset of the COVID-19 pandemic.

**Methods:**

Data collection was conducted in 2021 where participants completed an online questionnaire to assess probable major depressive disorders (MDD) using the 9-item Patient Health Questionnaire (PHQ-9).

**Results:**

Among the 655 respondents, 21.1% had probable MDD. Using log-binomial regression, we found an association between perceived loneliness, lack of psychosocial support at work and deterioration of one’s financial situation with probable depression. Further adjustment on mental health comorbidities revealed an association between both a deterioration of financial situation and living alone with probable depression.

**Conclusions:**

This study shown an association between social support and financial conditions and depressive symptoms among HCWs, regardless of level of education, pandemic and work-related factors and mental health comorbidities. This emphasises the need to improve working conditions for HCWs, combat their loneliness, increase wages and provide support for HCWs at high risk of depression.

**Disclosure of Interest:**

None Declared

